# Laparoscopy vs. Laparotomy for the Management of Abdominal Trauma: A Systematic Review and Meta-Analysis

**DOI:** 10.3389/fsurg.2022.817134

**Published:** 2022-03-08

**Authors:** Jianjun Wang, Liangwang Cheng, Jing Liu, Binyin Zhang, Weijun Wang, Wenxin Zhu, Yan Guo, Chuanfei Bao, Yunli Hu, Shanxin Qi, Kai Wang, Shuguang Zhao

**Affiliations:** ^1^Department of Emergency, Taihe People's Hospital, Fuyang, China; ^2^Department of Endocrine, Taihe People's Hospital, Fuyang, China

**Keywords:** abdominal trauma, laparoscopy, laparotomy, systematic review, meta-analysis

## Abstract

**Background:**

There is still no consensus regarding the role of laparoscopy in trauma cases. The purpose of this paper is to assess the value of diagnostic and therapeutic laparoscopy for patients with blunt or penetrating abdominal trauma by performing a systematic review and meta-analysis.

**Methods:**

PubMed, Embase, and the Cochrane library were systemically searched for the randomized controlled trials (RCTs) and non-RCT comparative studies on effectiveness and safety of laparoscopy vs. laparotomy for the two authors independently performed the search, data extraction, and quality assessment.

**Results:**

A total of 5,517 patients were enrolled in 23 eligible studies that were published in English. Meta-analysis results suggest that there is no significant difference in the incidence of missed injury and mortality between abdominal trauma patients receiving laparoscopy and those receiving laparotomy. Concerning postoperative complications, compared with patients in the open surgery group, those in the laparoscopy group are at a similar risk of intra-abdominal abscesses, thromboembolism, and ileus, while there is a decreased incidence of wound infection and pneumonia. Besides, patients in the laparoscopy group experience shorter hospitalization times and procedure times. For most outcomes, the sensitivity analysis yielded similar results to the primary analysis.

**Conclusion:**

Laparoscopic surgery is a practical alternative to laparotomy for appropriate patients. The decision to perform laparoscopy should be based on the experience of the surgeon and the resources available.

## Introduction

Trauma is the fourth leading cause of death in the overall population, while it is the main cause of death during the first half of the human life span (1). Besides, 9~14.9% of all trauma cases involve the abdomen (2). Abdominal trauma is one of the preventable causes of death in polytrauma patients (3), and laparotomy has traditionally been considered as the standard treatment (4). However, since laparotomy is associated with morbidity ranging from 20 to 40% (5–7), it may be preferable to avoid unnecessary laparotomies. In haemodynamically stable conditions and conducted by experienced surgeons, laparoscopy is an effective and safe in the management of abdominal trauma patients (8). Advances of imaging technology and selective non-operative management have led to a decrease in non-therapeutic laparotomy for haemodynamically stable patients (9–11). Studies have also shown that since the introduction of the laparoscopy procedure, the rate of non-therapeutic laparotomy has further decreased (7, 12). Moreover, as a diagnostic or therapeutic tool, laparoscopy involves less pain and results in a shorter hospital stays and faster recovery times than laparotomy. Although the feasibility and benefits of diagnostic and therapeutic laparoscopy in selected haemodynamically stable trauma patients have already been demonstrated, a widely accepted consensus has not yet been reached (8, 13). Soon after the laparoscopy procedure was introduced, several systematic reviews (14–16) summarizing its value for penetrating or blunt abdominal trauma were published. Subsequently, a series of papers (7, 8, 12, 17–21) were published addressing a wide range of possibilities for the application of laparoscopy in abdominal trauma. In this review, we integrated newly published studies with previous evidence to comprehensively compare the effectiveness and safety of laparoscopy with laparotomy on penetrating or blunt abdominal trauma.

## Methods

This study was conducted according to the Preferred Reporting Items for Systematic Review and Meta-Analyses (PRISMA) statement (22).

### Search Strategy

We searched PubMed, Embase, and the Cochrane Library for comparative studies on the effectiveness and safety of laparoscopy vs. laparotomy for the management of abdominal trauma up to 30^th^ June 31, 2021. The Medical Subject Headings (MeSH) including “laparoscopy,” “abdominal injuries,” as well as free text words like “laparoscop^*^,” “minimal^*^ invasive,” “abdom^*^,” “injur^*^,” “wound^*^,” “stab^*^,” “shot^*^,” “shoot^*^,” “lacerat^*^,” “trauma^*^,” “penetrat^*^,” and “blunt^*^,” in combination with the Boolean operators “AND” and “OR.” Besides, we also searched the references listed in all the articles that were initially selected. The Appendix in the Supplementary Materials gives details of the search strategies.

### Study Selection and Data Abstraction

Comparative studies that were published in English, and focused on the comparison of laparoscopy and laparotomy for the management of abdominal trauma were included. We excluded studies where the full text was not available, those that focused on children (age <18 years), and ones that did not select laparotomy as a comparator, or did not report on outcomes predefined in his review. The selection process of the relevant literature was conducted independently by two researchers (JW and LC), and any disagreements were resolved through discussion or by consulting a third author (SZ). Primary outcomes included missed injury, mortality, and postoperative complications such as wound infection, abscess formation, bowel obstruction or ileus, pneumonia, and thromboembolism. Additionally, the secondary outcomes encompassed procedure time, length of hospital stay, and re-operation. Two researchers (JW and LC) independently extracted the following information: (1) features of studies including the first author, published year, country, study design, study period, intervention, sample size, and rate of conversion to open surgery; (2) characteristics of patients including age, gender, injury severity score, abbreviated injury scale/ abdominal trauma index, and percentage of haemodynamic stable patients; (3) outcomes.

### Quality Assessment of the Eligible Studies

The bias risk of the eligible RCTs was assessed using Cochrane Collaboration's tool (23) for assessing the risk of bias in randomized trials, which includes the following seven domains: random sequence generation, allocation concealment, blinding of participants and personnel, blinding of outcome assessment, incomplete outcome data, selective reporting, and other bias. Each of the seven domains could be rated as having potentially high, unclear, or low risk of bias (24). The Newcastle-Ottawa Scale (NOS) was also applied to assess the quality of cohort studies. Three dimensions contributed to the overall quality score, including selection assessment of the exposed and unexposed cohort, comparability of the two cohorts, and outcome assessment. We graded the quality of cohort studies as high (≥8 stars), moderate (4–7 stars), or low (<4 stars), with a third person (SZ) resolving any disagreements.

### Statistical Analysis

Where possible, data analysis was based on the intention-to-treat principle for each study that was included, and we performed meta-analyses using Review Manager version 5.3.5 (Cochrane Collaboration) (25). We assessed the heterogeneity between studies using Cochran's Q test and the I^2^ statistics. Results were interpreted as either low (I2 = 0–40%), moderate (I2 = 30–60%), substantial (I2 = 50–90%) or high heterogeneity (I2 = 75–100%) (23, 26). We also calculated risk difference (RD) with a 95% confidence interval (CI) for the dichotomous outcomes, and the mean differences (MD) with 95% CI for continuous data. Subgroup analyses were performed according to the study design (prospective vs. retrospective), injury mechanism (penetrating vs. blunt abdominal trauma), and the purpose of laparoscopy use (diagnosis vs. treatment) for primary outcomes. A random effects model was applied in the primary analysis, whereas fixed effects models were used for sensitivity analysis. Furthermore, a sensitivity analysis was performed by removing the studies one by one. Additionally, meta-analyses were performed on only the high-quality studies. A qualitative description was performed for studies that were not suitable for quantitative data synthesis, and potential publication bias was assessed using funnel plots. *P* < 0.05 were considered statistically significant.

## Results

**Figure 1:**
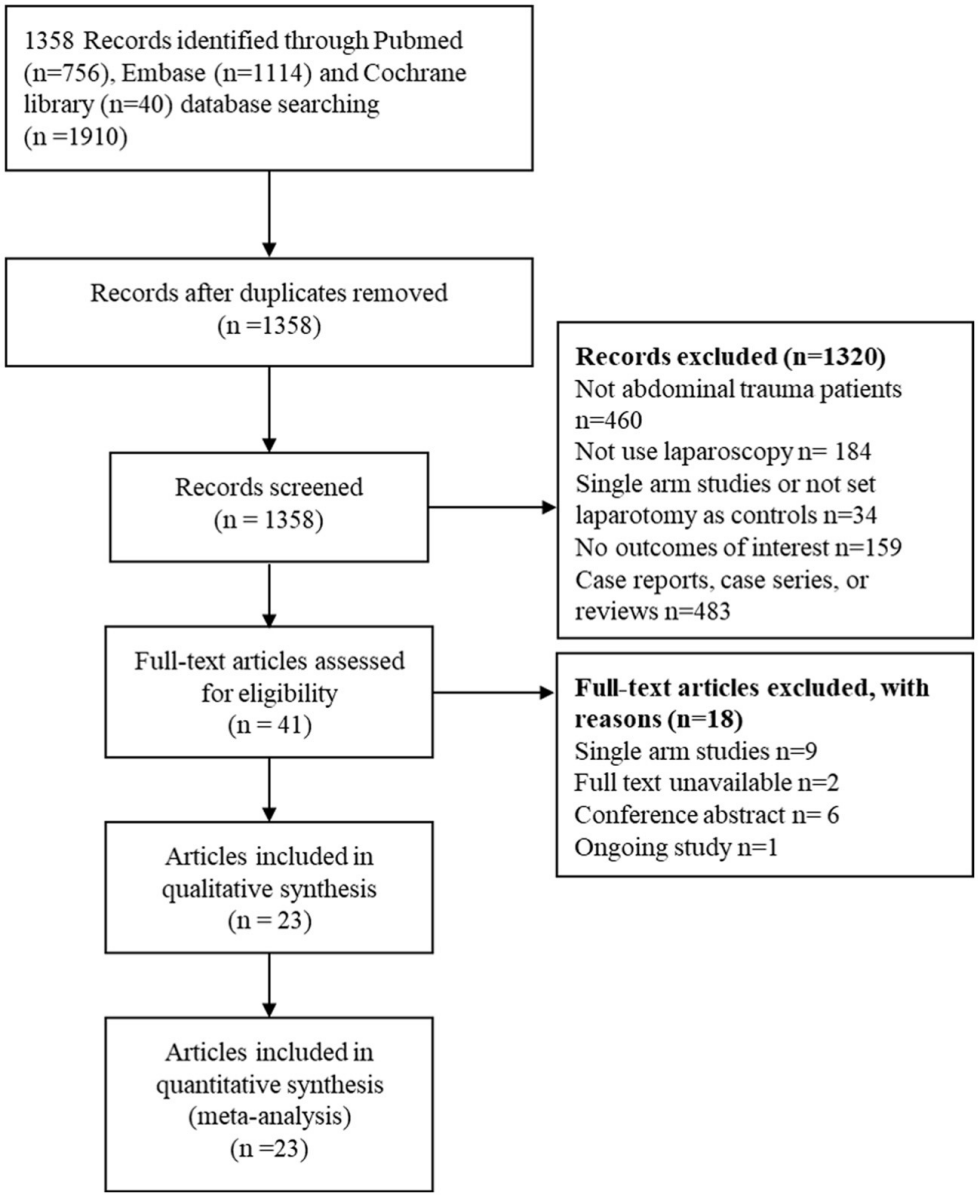
The PRISMA flow diagram for literature screening.

The literature search initially identified 1, 358 papers, of which 41 studies were eligible for the full-text screening process, and 23 studies (7, 8, 12, 17–21, 27–41) were included in the meta-analysis. The PRISMA flow diagram is illustrated in Figure 1.

The 23 studies encompassed one RCT (30), two prospective observational studies (29, 35) and 20 retrospective observational studies (7, 8, 12, 17–21, 27, 28, 31–34, 36–41). Four (33, 34, 37, 41) of the 20 retrospective observational studies were based on controlled before and after study designs, while the remainder were comparative/parallel studies. Nine (7, 8, 12, 18, 19, 21, 36, 39, 40) of the 23 studies were focussed on therapeutic laparoscopy or reported separately on diagnostic laparoscopy. A total of 5,517 individual patients were involved in the study, with 2,594 patients enrolling in the laparoscopy group, and 2,923 patients in the laparotomy group. All but one study (17) reported the conversion rate from laparoscopy to laparotomy, with an average value of 25.0% (range from 0 to 45.1%). The patient age varied from 26 to 57 years, with a 76.9% male population. Besides, the age did not differ significantly between the two intervention groups. Seventeen studies (7, 8, 12, 18, 19, 21, 27, 30–32, 34, 36–41) reported the severity of the condition evaluated by the injury severity score, new injury severity score, or abbreviated injury scale/abdominal trauma index, while the six other studies (17, 20, 28, 29, 33, 35) did not specify the severity scores at all. Additionally, in two studies, there was a significant difference in disease severity between the laparoscopy and laparotomy groups (12, 32). Almost all reports involved only haemodynamically stable patients, except for two studies (33, 39), which included a certain percentage of haemodynamically unstable patients. The basic characteristics of the studies and the patients involved in the meta-analysis are presented in Tables 1, 2.

**Table 1:** Characteristics of the included studies.

**References**	**Region**	**Study type**	**Study period**	**Intervention**	**Sample size**	**Conversion to open, ***n*** (%)**
Shams and Elyasi (17)	Iran	Retrospective observational study	1 year	Laparoscopy	18	NR
				Laparotomy	22	
Birindelli et al. (19)	Italy	Retrospective observational study	Jan 2013 to Dec 2017	Laparoscopy	16	3 (19.0%)
				Laparotomy	32	
Obaid et al. (18)	USA	Retrospective observational study	Jan 2017 to Dec 2017	Laparoscopy	177	13 (7.3%)
				Laparotomy	354	
Gao et al. (8)	China	Retrospective observational study	Jan 2013 to Dec 2017	Laparoscopy	54	4 (7.4%)
				Laparotomy	54	
Lin et al. (12)	Taiwan	Retrospective observational study	Jan 2006 to Dec 2015	Laparoscopy	126	9 (7.1%)
				Laparotomy	139	
Chakravartty et al. (7)	UK	Retrospective observational study	Jan 2004 to Jan 2014	Laparoscopy	25	1 (4.0%)
				Laparotomy	25	
Trejo-Ávila et al. (20)	Mexico	Retrospective observational study	Jan 2013 to May 2016	Laparoscopy	19	1 (5.3%)
				Laparotomy	19	
Huang et al. (21)	USA	Retrospective observational study	Jan 2011 to Dec 2014	Laparoscopy	11	0 (0%)
				Laparotomy	41	
Lim et al. (40)	South Korea	Retrospective observational study	Jan 2006 to Aug 2012	Laparoscopy	41	9 (18%)
				Laparotomy	55	
Chestovich et al. (39)	USA	Retrospective observational study	Jan 2008 to Dec 2013	Laparoscopy	94	15 (16.0%)
				Laparotomy	96	
Liao et al. (38)	Taiwan	Retrospective observational study	Jan 2010 to Jan 2013	Laparoscopy	15	1 (6.7%)
				Laparotomy	20	
Lee et al. (37)	Taiwan	Retrospective observational study	Jun 2003 to Jun 2006; Jul 2007 to Jun 2010	Laparoscopy	57	2 (3.5%)
				Laparotomy	47	
Karateke et al. (35)	Turkey	Prospective non-randomized study	Jun 2010 to Jul 2011	Laparoscopy	26	9 (34.6%)
				Laparotomy	26	
Khubutiya et al. (36)	Russia	Retrospective observational study	2000 to 2011	Laparoscopy	328	130 (37.3%)
				Laparotomy	280	
Lin et al. (34)	Taiwan	Retrospective observational study	Jan 1998 to Jan 2003; Jan 2003 to Dec 2007	Laparoscopy	48	1 (2.1%)
				Laparotomy	38	
Cherkasov et al. (33)	Russia	Retrospective observational study	1997 to 2003	Laparoscopy	1332	356 (26.7%)
				Laparotomy	1363	
Cherry et al. (32)	USA	Retrospective observational study	Jan1999 to Dec 2001	Laparoscopy	92	36 (39.1%)
				Laparotomy	64	
Miles et al. (31)	USA	Retrospective observational study	Jul 1999 to Jul 2002	Laparoscopy	22	9 (40.9%)
				Laparotomy	154	
Omori et al. (41)	Japan	Retrospective observational study	Jan1993 and Dec1997; Jan 1998 to Jan 2000	Laparoscopy	11	1 (9.1%)
				Laparotomy	13	
Leppäniem and Haapiainen (30)	Finland	RCT	May 1997 to Jan 2002	Laparoscopy	20	9 (45.0%)
				Laparotomy	23	
DeMaria et al. (29)	USA	Prospective observational study	Nov 1991 to Sep 1993	Laparoscopy	31	14 (45.1%)
				Laparotomy	23	
Mutter et al. (28)	France	Retrospective observational study	Feb 1990 to Jan 1996	Laparoscopy	17	4 (23.6%)
				Laparotomy	18	
Marks et al. (27)	USA	Retrospective observational study	Jan 1992 to Sep 1994	Laparoscopy	14	4 (28.6%)
				Laparotomy	19	

*NR, not report*.

**Table 2:** Characteristics of the included patients.

**References**	**Population**	**Intervention**	Age (y)*	**Male, ***n*** (%)**	**ISS**	**New ISS**	**AIS/ATI**	**Haemodynamically stable (%)**
Shams and Elyasi (17)	PAT	Laparoscopy	33.4 ± 15.1	26 (65.0)	NR	NR	NR	100.0%
		Laparotomy	27.8 ± 7.9		NR	NR	NR	100.0%
Birindelli et al. (19)	Splenic trauma	Laparoscopy	Mean 47	10 (62.5)	24	NR	NR	100.0%
		Laparotomy	Mean 50	22 (68.8)	20	NR	NR	100.0%
Obaid et al. (18)	TDI	Laparoscopy	36 ± 17	136 (76.8)	17 (10–22)	NR	2 (1–3)	100.0%
		Laparotomy	35 ± 16	280 (79.1)	17 (9–21)	NR	2 (1–2)	100.0%
Gao et al. (8)	PAT, BAT	Laparoscopy	39.1 ± 15.3	41 (75.9)	5.39 ± 2.72	NR	NR	100.0%
		Laparotomy	42.5 ± 13.6	42 (77.8)	4.67 ± 2.56	NR	NR	100.0%
Lin et al. (12)	BAT	Laparoscopy	38.5 ± 18.0	80 (63.5)	18.9 ± 8.5	NR	3.3 ± 0.6	100.0%
		Laparotomy	35.2 ± 16.2	99 (71.2)	23.3 ± 9.9	NR	3.7 ± 0.7	100.0%
Chakravartty et al. (7)	AT	Laparoscopy	33 (14–62)	21 (84.0)	16 (4–34)	NR	NR	100.0%
		Laparotomy	26 (16–58)	23 (92.0)	16 (3–29)	NR	NR	100.0%
Trejo-Ávila et al. (20)	PAT, BAT	Laparoscopy	25.5 ± 7.7	17 (89.5)	NR	NR	NR	100.0%
		Laparotomy	30.9 ± 10.9	19 (100.0)	NR	NR	NR	100.0%
Huang et al. (21)	BAT	Laparoscopy	Mean 47.2	6 (54.5)	Mean 21.6	NR	Mean 3.4	100.0%
		Laparotomy	Mean 49.1	30 (73.2)	Mean 28.6	NR	Mean 3.8	100.0%
Lim et al. (40)	AT	Laparoscopy	53.8 ± 15.7	NR	9.3 ± 3.6	NR	3.2 ± 1.4	100.0%
		Laparotomy	57.2 ± 15.6		9.1 ± 2.8	NR	3.2 ± 0.9	100.0%
Chestovich et al. (39)	PAT	Laparoscopy	28 (22–42)^a^/ 29 (23–37)^b^	82 (87.2)	1 (1–3)^a^/ 1 (1–3)^b^	1 (1–3)^a^/9 (4–15)^b^	1 (1–1)^a^/ 2 (2–3)^b^	100.0%
		Laparotomy	31 (23–42)^a^/ 30 (22–40)^b^	88 (91.7)	8 (4–13) ^a^/ 9 (4–12)^b^	1 (1–3)^a^/ 13 (5–20)^b^	1 (1–1)^a^/ 3 (2–3)^b^	97.9%
Liao et al. (38)	PAT, BAT	Laparoscopy	44.4 ± 13.8	10 (66.7)	11.5 ± 6.7	NR	NR	100.0%
		Laparotomy	44.1 ± 16.2	25 (83.3)	11.8 ± 5.1	NR	NR	100.0%
Lee et al. (37, 38)	BAT	Laparoscopy	38.0 ± 19.4	37 (64.9)	17.6 ± 8.2	NR	NR	100.0%
		Laparotomy	33.6 ± 15.9	37 (78.7)	20.2 ± 6.9	NR	NR	100.0%
Karateke et al. (35)	PAT	Laparoscopy	33.2 ± 9.2	45 (86.5)	NR	NR	NR	100.0%
		Laparotomy	35.2 ± 10.6		NR	NR	NR	100.0%
Khubutiya et al. (36)	PAT, BAT	Laparoscopy	34.5 ± 14^c^/ 35.8 ± 3.5^d^	273 (78.4)	14.6 ± 0.7^c^/ 9.8 ± 0.5^d^	NR	NR	100.0%
		Laparotomy	33.5 ± 2.5^c^/ 36.5 ± 2.3^d^	220 (78.6)	14.9 ± 0.7^c^ /6.4 ± 0.2^d^	NR	NR	100.0%
Lin et al. (34)	abdominal stab wounds	Laparoscopy	41.1 ± 14.3	NR	4.3 ± 4.8	NR	3.9 ± 4.7	100.0%
		Laparotomy	43.8 ± 11.6	NR	5.7 ± 5.0	NR	5.1 ± 5.5	100.0%
Cherkasov et al. (33)	PAT	Laparoscopy	NR	NR	NR	NR	NR	8.7% of total patients
		Laparotomy	NR	NR	NR	NR	NR	
Cherry et al. (32)	PAT	Laparoscopy	29.4 ± 1.2	NR	5.5 ± 0.6	NR	NR	100.0%
		Laparotomy	29.1 ± 1.4	NR	9.0 ± 0.8	NR	NR	100.0%
Miles et al. (31)	PAT	Laparoscopy	Mean 32.8	171 (81.8)	Mean 13.6	NR	NR	100.0%
		Laparotomy			Mean 6.4	NR	NR	100.0%
Omori et al. [41	BAT	Laparoscopy	50.6 ± 18.2	8 (72.7)	11.8 ± 5.8	NR	NR	100.0%
		Laparotomy	45.9 ± 13.6	9 (69.2)	14.4 ± 5.7	NR	NR	100.0%
Leppäniemi and Haapiainen (30)	Stab wounds	Laparoscopy	39 ± 11	16 (85)	6 ± 3	8 ± 6	9 ± 9	100.0%
		Laparotomy	41 ± 13	21 (91)	8 ± 5	9 ± 7	6 ± 6	100.0%
DeMaria et al. (29)	Abdominal stab wounds	Laparoscopy	NR	NR	NR	NR	NR	100.0%
		Laparotomy	NR	NR	NR	NR	NR	100.0%
Mutter et al. (28)	Abdominal stab wounds	Laparoscopy	34 (17–62)	32 (91.4)	NR	NR	NR	100.0%
		Laparotomy			NR	NR	NR	100.0%
Marks et al. (27)	PAT	Laparoscopy	30.5 ± 2.4	NR	2.4 ± 0.6	NR	NR	100.0%
		Laparotomy	31.2 ± 2.2	NR	3.2 ± 0.7	NR	NR	100.0%

*PAT, penetrating abdominal trauma; BAT, Blunt abdominal trauma; TDI, Traumatic diaphragmatic injury; ISS, injury severity score; AIS, abbreviated injury scale; ATI, abdominal trauma index; NR, not reported*.

* *Presented as mean ± standard deviation, median (range) or median (interquartile range)*.

a *Diagnostic laparoscopy*.

b *Therapeutic laparoscopy*.

c *Blunt abdominal trauma*.

d *PAT, penetrating abdominal trauma*.

### Quality Assessment of the Included Studies

Although the only RCT (30) we reviewed did not implement the blinding method, the author specified random sequence generation and allocation concealment. With respect to incomplete outcome data and selective reporting, the RCT demonstrated a low risk of bias. Since blinding was not possible due to the nature of the surgical interventions, we deemed this RCT as a high-quality study. The quality of the selected cohort studies assessed by the NOS was moderate-to-high, although around half of the included studies (12, 17, 21, 28, 29, 31–33, 35, 36) did not describe in detail the methods used to avoid bias in the comparability domain. The details of the quality assessment are presented in Supplementary Tables S1, S2.

### Meta-Analysis Results

#### Missed Injury

**Figure 2:**
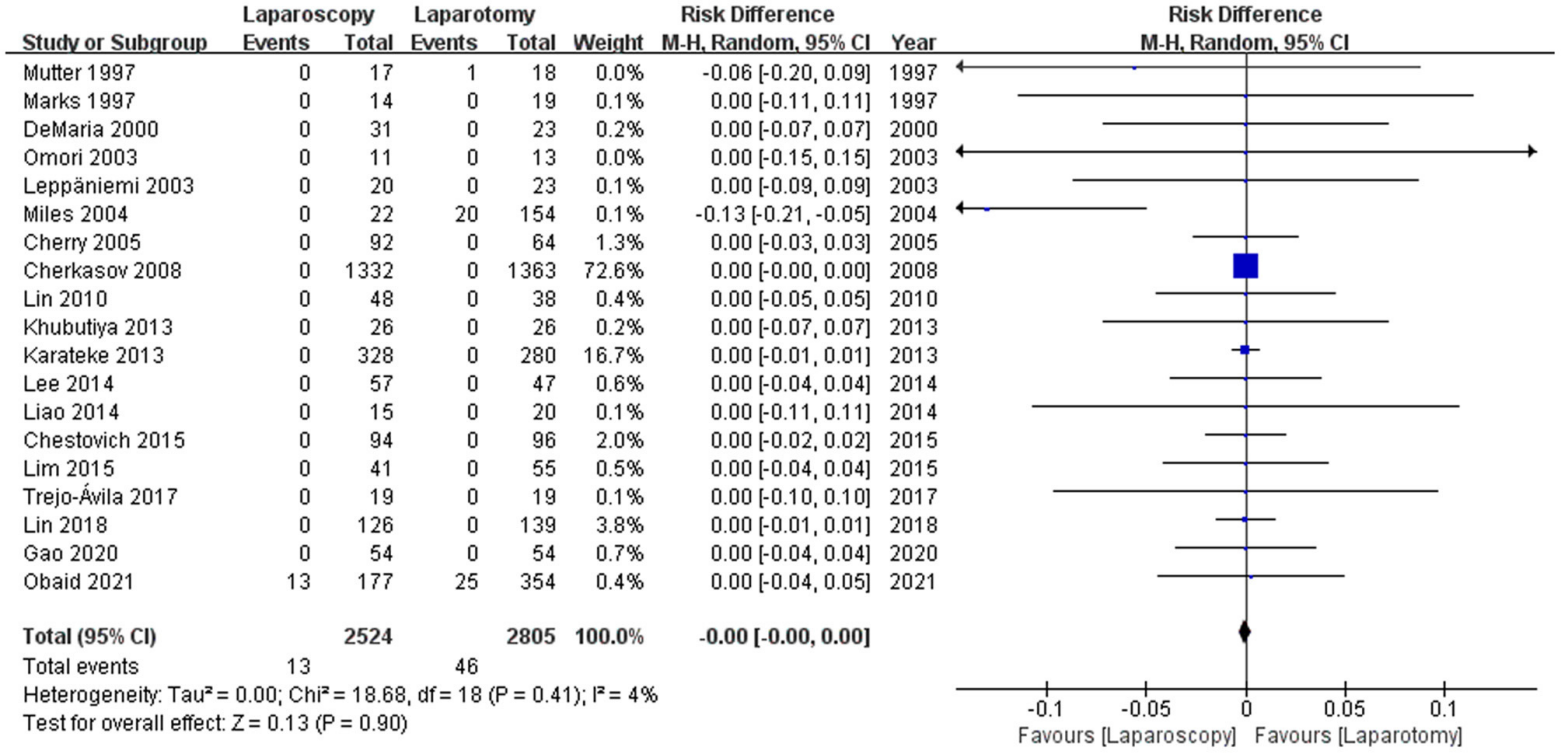
Forest plot of the comparison of laparoscopy vs. laparotomy for abdominal trauma, outcome: missed injury. M-H, Mantel-Haenszel; CI, confidence interval.

A total of 19 studies (8, 12, 18, 20, 27–35, 37–41) (5,327 patients) reported the cases of missed injury. In the laparoscopy arm, which resulted in 13 missed injuries in the laparoscopy group and 46 in the laparotomy group. The difference between the two groups (0.52vs 1.64%) was not statistically significant [RD −0.00, 95%CI (−0.00, 0.00), *p* = 0.90] (see Figure 2). The I2 statistic for heterogeneity among studies was 4%, suggesting low heterogeneity.

#### Mortality

**Figure 3:**
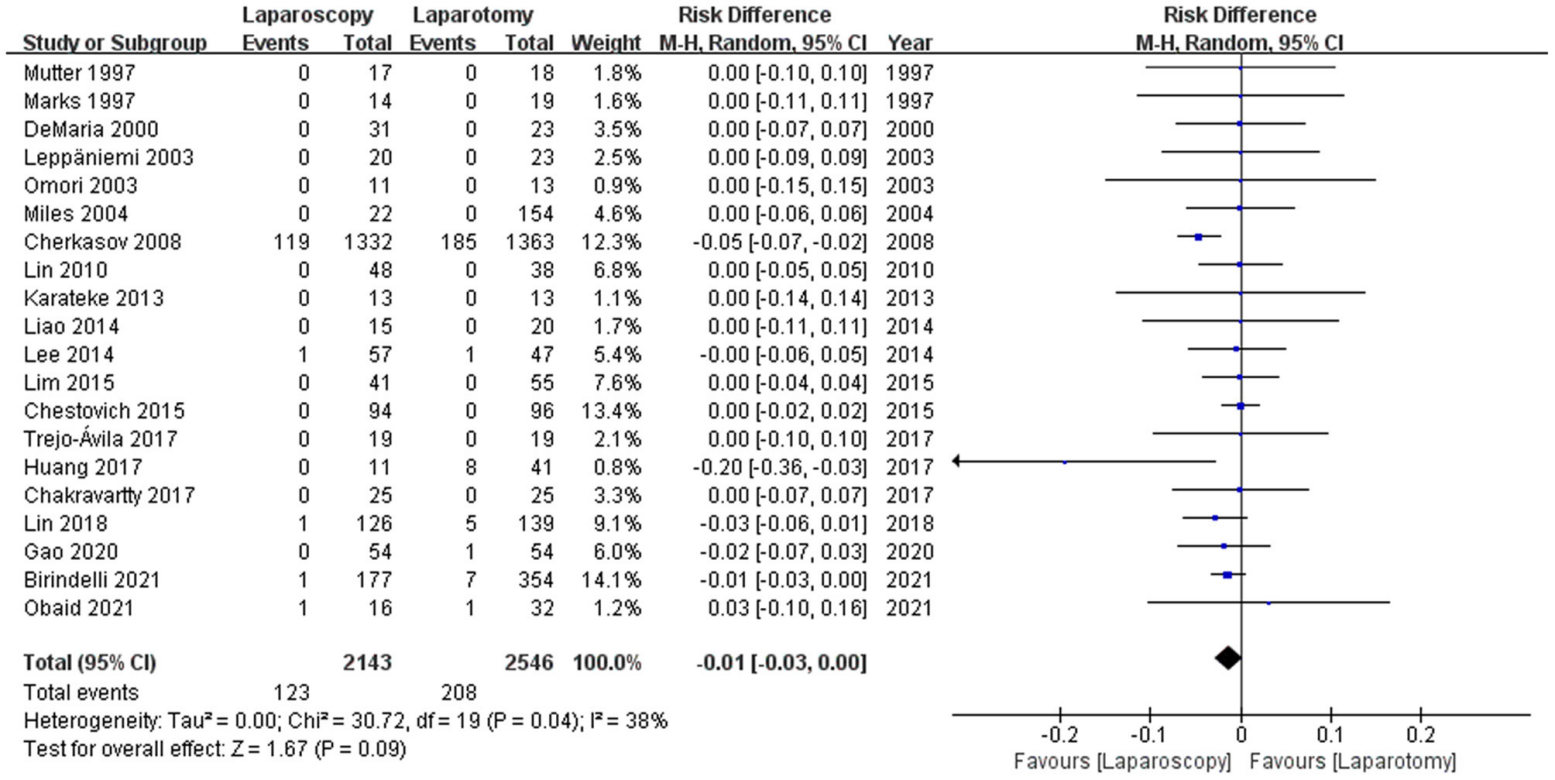
Forest plot of the comparison of laparoscopy vs. laparotomy for abdominal trauma, outcome: mortality. M-H, Mantel-Haenszel; CI, confidence interval.

Of the 23 studies, 20 (8, 12, 18–21, 27–31, 33–35, 37–41) reported mortalities. Overall, there were 123 mortalities in the laparoscopy group and 208 in the laparotomy group. There was no significant disparity in the incidence of mortality between the groups [5.74 vs. 8.17%, RD −0.01, 95%CI (−0.03, 0.00), *p* = 0.09], with moderate heterogeneity (I2 = 38%; see Figure 3).

#### Wound Infection

**Figure 4:**
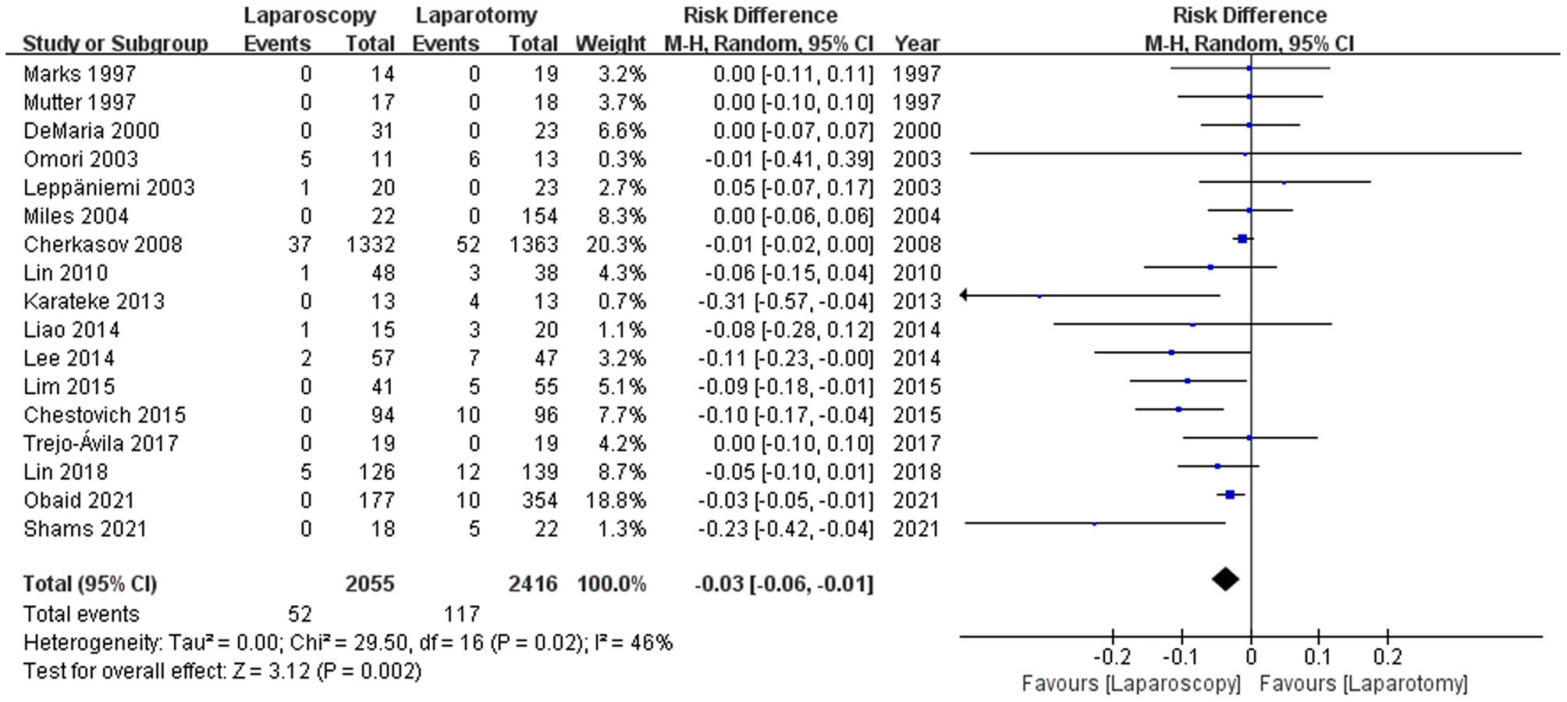
Forest plot of the comparison of laparoscopy vs. laparotomy for abdominal trauma, outcome: wound infection. M-H, Mantel-Haenszel; CI, confidence interval.

Regarding complications, wound infections were reported by 17 studies (12, 17, 18, 20, 27–31, 33–35, 37–41), and the wound infection rate was 52 of 2055 (2.53%) patients in the laparoscopy group and 117 of 2,416 (4.84%) patients in the laparotomy group. Patients who underwent laparoscopy had a substantially lower incidence of wound infection than those in the laparotomy group [RD −0.03, 95%CI (−0.06, −0.01), *p* = 0.002] (see Figure 4). Heterogeneity among the studies was moderate (I2 = 46%).

#### Intra-Abdominal Abscess

**Figure 5:**
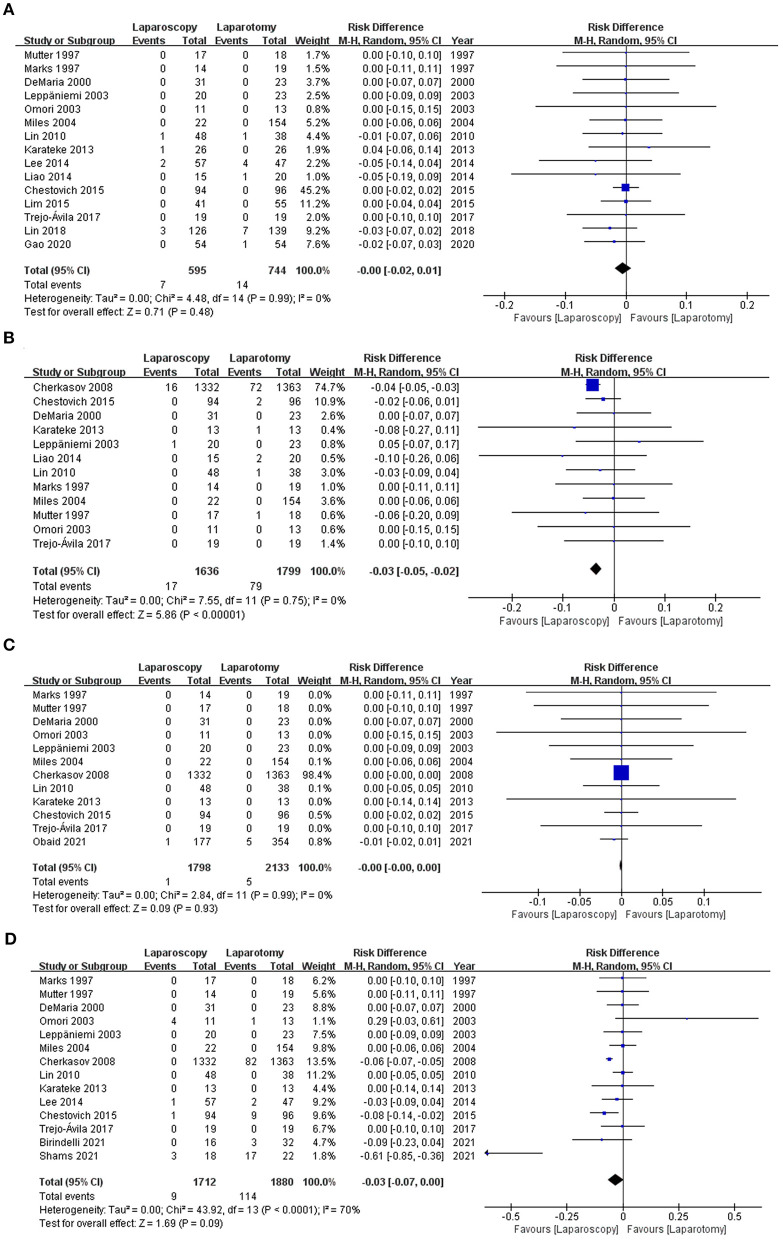
Forest plot of the comparison of laparoscopy vs. laparotomy for abdominal trauma, outcome: **(A)** intra-abdominal abscess; **(B)** pneumonia; **(C)** thromboembolism; **(D)** bowel obstruction or ileus. M-H, Mantel-Haenszel; CI, confidence interval.

Of the 23 studies, 15 (8, 12, 20, 27–31, 34, 35, 37–41) of them including 1,339 patients, evaluated intra-abdominal abscesses. Seven patients treated with laparoscopy developed abscesses, compared with 14 patients in the laparotomy group. Both groups had similar rates of abscesses [1.18 vs. 1.88%, RD −0.00, 95% CI (−0.02, 0.01), *p* = 0.48] (see Figure 5A), and there was no significant heterogeneity across the studies (I2 = 0%).

#### Pneumonia

Twelve studies (20, 27–31, 33–35, 38, 39, 41), involving 1,636 patients in the laparoscopy group and 1,799 patients in the laparotomy group, investigated the incidence of pneumonia. Pneumonia occurred 17 times within the laparoscopy group and 79 times in the laparotomy group, indicating a substantially higher incidence rate for patients who underwent laparotomy [1.04 vs. 4.39%, RD −0.03, 95% CI (−0.05, 0.02), *p* < 0.00001]. There was no statistical heterogeneity among the studies (I2 = 0%; see Figure 5B).

#### Thromboembolism

Twelve studies (18, 20, 27–31, 33–35, 39, 41) examined thromboembolism, and only one reported that thromboembolism was occurred in both groups. The pooled analysis indicated that the proportion of thromboembolism was comparable between the two groups [0.05 vs. 0.23%, RD −0.00, 95% CI (−0.00, −0.00), *p* = 0.93], and there was no heterogeneity (I2 = 0%; see Figure 5C).

#### Bowel Obstruction or Ileus

There were 14 studies (17, 19, 20, 27–31, 33–35, 37, 39, 41) that reported data on bowel obstruction or ileus. Compared to patients in the laparotomy group, there was a lower incidence of ileus in the laparoscopy group (0.53 vs. 6.06%). However, the difference was not statistically significant in a pooled analysis [RD −0.03, 95% CI (−0.07, −0.00), *p* = 0.09]. Heterogeneity analyses suggested substantial heterogeneity across the studies (I2 = 70%; see Figure 5D).

#### Length of Stay

**Figure 6:**
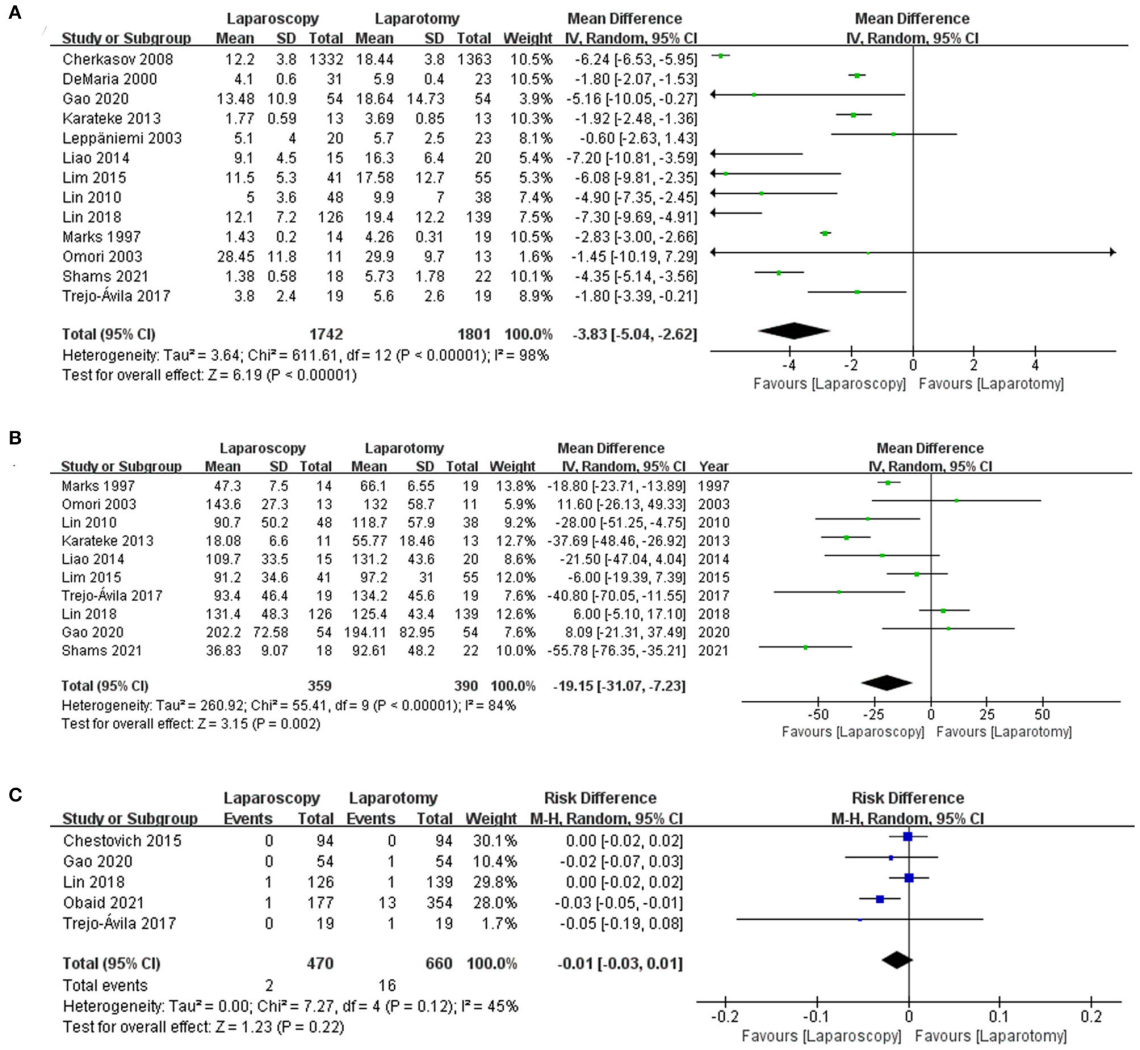
Forest plot of the comparison of laparoscopy vs. laparotomy for abdominal trauma, outcome: **(A)** the length of stay; **(B)** procedure time; **(C)** re-operation. IV, Inverse variance; CI, confidence interval.

All of the included studies considered the length of stay of patients with abdominal trauma. However, only 13 studies (8, 12, 17, 20, 27, 29, 30, 33–35, 38, 40, 41) of them were included in the quantitative synthesis. Meta-analysis results suggested that the length of stay of patients who underwent laparoscopy was significantly shorter than those who underwent laparotomy [MD −3.83, 95% CI (−5.04, −2.62) days, *p* < 0.00001], there was high heterogeneity (I2 = 98%; see Figure 6A). Eight (7, 18, 28, 31, 32, 36, 37, 39) of the ten remaining studies whose data was not suitable for quantitative synthesis were consistent with the above results, while the findings of two studies (19, 21) did not favor the above results.

#### Procedure Time

Of the 14 studies (8, 12, 17, 19–21, 27, 34, 35, 37, 38, 40, 41) that provided details of the procedure time, ten studies (8, 12, 17, 20, 27, 34, 35, 38, 40, 41) including 749 patients could be synthesized quantitatively. By comparing the operation conditions of two groups of patients, results from the meta-analysis showed that the procedure time of the laparoscopy was significantly shorter than laparotomy [MD −19.15, 95% CI (−31.07, −7.23) min, *p* = 0.002] (see Figure 6B). Nevertheless, heterogeneity among the studies was high (I2 = 84%). One (37) of the four studies that were performed qualitatively concurred with the above meta-analysis results. However, the three remaining studies (7, 19, 21) suggested that patients in laparoscopy group experienced a significantly longer procedure time compared with those in laparotomy.

#### Re-operation

Five studies (8, 12, 18, 20, 39) comprising 1,130 patients reported the rate of re-exploration, and the pooled analysis indicated that the rate of re-exploration did not differ significantly between the two groups [0.43 vs. 2.42%, RD −0.01, 95% CI (−0.03, 0.01), *p* = 0.22]. Besides, there was moderate heterogeneity (I2 = 45%; see Figure 6C).

#### Subgroup Analyses and Sensitivity Analyses

There was no statistical difference between subgroups based on study design, injury mechanism, and the purpose of laparoscopy use for primary outcomes (see Supplementary Tables S3–S5). Using fixed effects models did not substantially alter the results of any outcomes except for mortality, ileus, and re-operation (see Supplementary Table S6). Besides, sensitivity analyses indicated that the pooled results of mortality and pneumonia were varied after excluding the studies one by one. Concerning mortality, the results changed significantly when the study of Chestovich et al. (39) was removed [6.00 vs. 8.49%, RD −0.02, 95% CI (−0.03, −0.00), *p* = 0.02, I2 = 19%]. Moreover, the heterogeneity disappeared after removing the study of Cherkasov et al. (33) (from 41 to 0%), without causing a significant change in the pooled estimate. Besides, the results became statistically insignificant in the meta-analysis of pneumonia [0.33 vs. 1.61%, RD −0.01, 95% CI (−0.04, −0.01), *p* = 0.20] after removing the study of Cherkasov et al. (33), although there was still a lower trend toward laparoscopy. Also, inter-study heterogeneity was insignificant (I2 = 0%). After removing the study of Shams and Elyasi (17), the inter-study heterogeneity fell significantly (from 71 to 44%) for ileus. Additionally, the pooled results of high-quality studies (7, 8, 18–20, 27, 34, 37–40) were consistent with the results of the primary analyses for all outcomes except pneumonia (see Supplementary Table S7). Here, the difference in the risk of pneumonia became insignificant in the comparison of laparoscopy vs. laparotomy [0.48 vs. 2.35%, RD −0.02, 95% CI (−0.05, −0.01), *p* = 0.20, I2 = 0%]. Unfortunately, the sensitivity analyses did not reveal the source of high heterogeneity of length of stay and procedure time.

#### Publication Bias

The funnel plot of each outcome assessing the risk of publication bias showed symmetric distribution, indicating an absence of publication bias.

## Discussion

The results of this study demonstrate that there is no significant difference in the incidence of missed injury and mortality between abdominal trauma patients receiving laparoscopy and those receiving laparotomy. Regarding postoperative complications, compared with patients in the laparotomy group, patients in the laparoscopy group have a similar risk of undergoing re-exploration or developing intra-abdominal abscesses, thromboembolism, and ileus, but there is a decreased incidence of wound infection and pneumonia. Besides, patients in the laparoscopy group experienced shorter hospitalization and procedure times.

Our results are largely consistent with previous reviews (15, 16). However, it was worth mentioning that there were two advantages in our study. First, we added several pieces of evidence from literature and comprehensively analyzed the value of diagnostic and therapeutic laparoscopy for patients with penetrating vs. blunt abdominal trauma. Moreover, this study conducted detailed subgroup analyses and found that there was no statistical difference between subgroups based on study design, injury mechanism, and the purpose of laparoscopy use. Finally, the findings demonstrated that therapeutic laparoscopy can serve as a safe and effective alternative in hemodynamically stable patients with abdominal trauma.

In this review, about one-quarter of all patients who had been recommended for laparoscopy needed to convert to laparotomy, with various conversion rates in all of the included studies. This is probably because the ability to conduct laparoscopy depends on hospital resources and the surgical skills of the surgeon (20). Another factor that may influence the results is that, different hospitals adopt various policies, and some centers recommend routine open surgery while others conduct laparoscopy in comparable patients (4, 7, 42, 43). Additionally, we noted that conversion rates are lower now than they were a decade ago, perhaps due to technological improvements in laparoscopic instruments and the accumulation of procedural experience. Also, with the advance in both laparoscopic experience and surgical techniques, the rate of missed injury has declined from 13 to 0.12%, a similar rate to its open surgery counterpart (44, 45). In this review, we calculated the overall rate of missed injury was 0.52% in the laparoscopy group, which was lower than the 3.2% reported by a review published in 2013 (46).

Laparoscopy benefits patients by significantly reducing peri-operative complications and hospital stays, improving quality of life, and accelerating their return to normal activities. Wound infection was the most commonly encountered complication in this review, with an overall incidence of 2.53% in the laparoscopy group, which was far lower than laparotomy. This is consistent with other studies that showed fewer wound infections following laparoscopic procedures, such as appendectomy (47) and cholecystectomy (48, 49). This could be due to the reduced surgical stress and tissue trauma that is imposed on the patient as a result of the minimally invasive approach. Multiple factors have been reported to be involved in this process, including less surgical trauma, a smaller incision, earlier mobilization, less postoperative pain, a less pronounced proinflammatory response than open surgery, and better preservation of the systemic immune function (50–52). The decreased incidence of pneumonia in patients with laparoscopy should be taken cautiously because of the inconsistencies between primary and sensitivity analysis. The inclusion of Cherkasov's study (33) may be responbile for the significant results. In the study (33), only 8% of the patients were haemodynamically stable, indicating that laparoscopy seemed to reduce the incidence of pneumonia in haemodynamically unstable patients. Also, sensitivity analyses did not support the robustness of results of the primary analyses for incidence of ileus. Given the significant heterogeneity between studies, the random-effects model was more appropriate because it provided a more conservative and reliable estimate of pooled RD. Coupled with the negative results of the pooled analysis of high-quality studies, we believed that there is no significant difference between the two surgical modes concerning the incidence of ileus. Finally, we found that laparoscopy is related to a decrease in the length of stay by approximately 4 days, and was close to the value of 5 days reported in the previous systematic review (15). Although there was high heterogeneity among the included studies, and we were not able to identify the source of heterogeneity, we still considered that the results were credible due to the consistency across all sensitivity analyses.

There are several limitations in this study that should be highlighted. First, only one of the studies included in our review was an RCT, and a majority of the studies were retrospective. These studies inherently contain a greater potential for misinterpretations than RCTs. However, because of the small number of trauma patients requiring surgical intervention, designing prospective comparative studies or RCTs may be difficult from an ethical or logistical perspective (7). Moreover, several studies included in this review were from historical cohorts of abdominal trauma, and the management methods do not correspond with current practice. Nonetheless, our study was not powered to see differences between the subgroups stratified by study designs. Second, some outcomes were not clearly defined, such as the differentiation between wound infection and intra-abdominal abscess. If a study reported organ space infection, that is technically an intra-abdominal abscess. However, there is no way to definitively distinguish between the two outcomes, so we could only assume that the determination of intra-abdominal abscess was correct when performing the quantitative synthesis. Finally, the experience of surgeon and trauma center infrastructure are important factors for assessing the laparoscopic operations, but a half of the included studies did not specify the experience of laparoscopic trauma surgeons and the volume of trauma centers. This makes it difficult to evaluate the performance bias by stratification.

## Conclusion

Laparoscopic surgery is a reasonable alternative to open surgery for the appropriate patients, but the intervention should be performed by the experienced surgeons in well-equipped health care facilities. However, more well-organized RCTs are required to verify the value of laparoscopy for diagnosing and treating abdominal trauma.

## Data Availability Statement

The original contributions presented in the study are included in the article/Supplementary Material, further inquiries can be directed to the corresponding author/s.

## Author Contributions

SZ and KW proposed the conception or study design. JW and LC performed literature search and screening and risk of bias assessment. JW wrote the manuscript. JL, BZ, WW, WZ, YG, and CB were responsible for data analysis. YH and SQ revised the manuscript. All authors agree to be responsible for all aspects of the work and finally approved the version to be published.

## Funding

This study was supported by Foundation of Emergency Medical Association of Anhui Province (FY2019-098).

## Conflict of Interest

The authors declare that the research was conducted in the absence of any commercial or financial relationships that could be construed as a potential conflict of interest.

## Publisher's Note

All claims expressed in this article are solely those of the authors and do not necessarily represent those of their affiliated organizations, or those of the publisher, the editors and the reviewers. Any product that may be evaluated in this article, or claim that may be made by its manufacturer, is not guaranteed or endorsed by the publisher.
