# Supplementary material for: Laparoscopy vs. Laparotomy for the Management of Abdominal Trauma: A Systematic Review and Meta-Analysis
Source: Front Surg. 2022 Mar 8;9:817134. doi: 10.3389/fsurg.2022.817134 (PMC8957831; doi:10.3389/fsurg.2022.817134)
Supplement: Supplementary file 1 [file Data_Sheet_1.docx]

**Supplementary materials**

**Caption**

**Appendix** Search strategy

**Table S1** Assessment of risk of bias of the randomized trial using the Cochrane Collaboration’s tool

**Table S2** Assessment of risk of bias of the cohort studies using the Newcastle-Ottawa Scale

**Table S3** Subgroups analyses results of missed injury

**Table S4** Subgroups analyses results of mortality

**Table S5** Subgroups analyses results of complications

**Table S6** The sensitive analysis using fixed effects model

**Table S7** The pooled analysis results of high-quality studies

**Appendix Search strategy**

**Pubmed**

((("Abdominal Injuries"[Mesh]) OR ((Abdom*[Title/Abstract]) AND (((((((injur*[Title/Abstract]) OR (trauma*[Title/Abstract])) OR (wound*[Title/Abstract])) OR (stab*[Title/Abstract])) OR (shot*[Title/Abstract])) OR (shoot*[Title/Abstract])) OR (lacerat*[Title/Abstract])))) AND ((penetrat*[Title/Abstract]) OR (blunt*[Title/Abstract]))) AND ((("Laparoscopy"[Mesh]) OR (Laparoscop*[Title/Abstract])) OR (minimal* invasive[Title/Abstract]))

**Embase**

#19 #15 AND #18

#18 #16 OR #17

#17 blunt*:ti,ab,kw

#16 penetrat*:ti,ab,kw

#15 #13 AND #14

#14 #2 OR #3 OR #4 OR #12

#13 #1 OR #5 OR #6 OR #7 OR #8 OR #9 OR #10 OR #11

#12 (minimally NEAR/3 invasive):ti,ab,kw

#11 (abdom* NEAR/3 lacerat*):ti,ab,kw

#10 (abdom* NEAR/3 shoot*):ti,ab,kw

#9 (abdom* NEAR/3 shot*):ti,ab,kw

#8 (abdom* NEAR/3 stab*):ti,ab,kw

#7 (abdom* NEAR/3 wound*):ti,ab,kw

#6 (abdom* NEAR/3 trauma*):ti,ab,kw

#5 (abdom* NEAR/3 injur*):ti,ab,kw

#4 'minimally invasive procedure'/exp

#3 'laparoscop*':ti,ab,kw

#2 'laparoscopy'/exp

#1 'abdominal injury'/exp

**Cochrane Library**

ID Search

#1 MeSH descriptor: [Abdominal Injuries] explode all trees

#2 Abdominal:ti,ab,kw

#3 (injur* OR trauma* OR wound* OR stab* OR shot* OR shoot* OR lacerat*):ti,ab,kw

#4 #2 AND #3

#5 #1 OR #4

#6 MeSH descriptor: [Laparoscopy] explode all trees

#7 laparoscop*:ti,ab,kw

#8 minimally invasive:ti,ab,kw

#9 #6 OR #7 OR #8

#10 #5 AND #9 in Trials

**Table S1 Assessment of risk of bias of the randomized trial using the Cochrane Collaboration’s tool**

| **Domains** | **Leppäniemi et al.(31), 2003** | |
| --- | --- | --- |
|  | **Authors’ judgment** | **Support for judgment** |
| Random sequence generation (selection bias) | Low risk | Details of randomisation reported |
| Allocation concealment (selection bias) | Low risk | Details of allocation concealment reported |
| Blinding of participants and personnel (performance bias) | Unclear risk | Blinding was not possible due to nature of surgical interventions |
| Blinding of outcome assessment (detection bias) | Low risk | Unlikely considering the fact that the outcome measures used being objective in nature |
| Incomplete outcome data (attrition bias) | Low risk | No loss to follow-up due to small numbers and short time scale (all patients fully reported) |
| Selective outcome reporting (reporting bias) | Low risk | Most major outcome have been reported. |
| Other potential threats to validity | Low risk | Similar baseline characteristics in both groups. No conflicts of interest reported |

**Table S2 Assessment of risk of bias of the cohort studies using the Newcastle-Ottawa Scale**

| Author | Year | Selection | | | | Comparability | Outcome | | | Total score |
| --- | --- | --- | --- | --- | --- | --- | --- | --- | --- | --- |
|  |  | Representativeness of the exposed cohort | Selection of the non exposed cohort | Ascertainment of exposure | Demonstration that outcome of interest was not present at start of study | Comparability of cohorts on the basis of the design or analysis | Assessment of outcome | Was follow-up long enough for outcomes to occur | Adequacy of follow up of cohorts |  |
| Shams(18) | 2021 | 1 | 1 | 1 | 1 | 0 | 1 | 1 | 1 | 7 |
| Birindelli(20) | 2021 | 1 | 1 | 1 | 1 | 1 | 1 | 1 | 1 | 8 |
| Obaid(19) | 2021 | 1 | 1 | 1 | 1 | 2 | 1 | 1 | 1 | 9 |
| Gao(9) | 2021 | 1 | 1 | 1 | 1 | 2 | 1 | 1 | 1 | 9 |
| Lin(13) | 2018 | 1 | 1 | 1 | 1 | 0 | 1 | 1 | 1 | 7 |
| Chakravartty(8) | 2017 | 1 | 1 | 1 | 1 | 1 | 1 | 1 | 1 | 8 |
| Trejo-Ávila(21) | 2017 | 1 | 1 | 1 | 1 | 1 | 1 | 1 | 1 | 8 |
| Huang(22) | 2017 | 1 | 1 | 1 | 1 | 0 | 1 | 1 | 1 | 7 |
| Lim(41) | 2015 | 1 | 1 | 1 | 1 | 1 | 1 | 1 | 1 | 8 |
| Chestovich(40) | 2015 | 1 | 1 | 1 | 1 | 2 | 1 | 1 | 1 | 9 |
| Liao(39) | 2014 | 1 | 1 | 1 | 1 | 2 | 1 | 1 | 1 | 9 |
| Lee(38) | 2014 | 1 | 1 | 1 | 1 | 1 | 1 | 1 | 1 | 8 |
| Karateke(36) | 2013 | 1 | 1 | 1 | 1 | 0 | 1 | 1 | 1 | 7 |
| Khubutiya(37) | 2013 | 1 | 1 | 1 | 1 | 0 | 1 | 1 | 1 | 7 |
| Lin(35) | 2010 | 1 | 1 | 1 | 1 | 1 | 1 | 1 | 1 | 8 |
| Cherkasov(34) | 2008 | 0 | 1 | 1 | 1 | 0 | 1 | 1 | 1 | 6 |
| Cherry(33) | 2005 | 1 | 1 | 1 | 1 | 0 | 1 | 1 | 1 | 7 |
| Miles(32) | 2004 | 1 | 0 | 1 | 1 | 0 | 1 | 1 | 1 | 6 |
| Omori(42) | 2003 | 1 | 1 | 1 | 1 | 1 | 1 | 1 | 1 | 8 |
| DeMaria(30) | 2000 | 1 | 1 | 1 | 1 | 0 | 1 | 1 | 1 | 7 |
| Mutter(29) | 1997 | 0 | 1 | 1 | 1 | 0 | 1 | 1 | 1 | 6 |
| Marks(28) | 1997 | 1 | 1 | 1 | 1 | 1 | 1 | 1 | 1 | 8 |

**Table S3** **Subgroups analyses results of missed injury**

| **Subgroup** | **Studies (n)** | **Risk Difference (95% CI)** | **P value between groups** |
| --- | --- | --- | --- |
| **Study design** |  |  | 0.91 |
| Prospective study | 3 | 0.00 (-0.01, 0.01) |  |
| Retrospective parallel control study | 12 | -0.00 (-0.01, 0.01) |  |
| Retrospective before-after study | 4 | 0.00 (-0.00, 0.00) |  |
| **Injury mechanism** |  |  | 0.79 |
| PAT | 7 | -0.01 (-0.03, 0.01) |  |
| BAT | 3 | 0.00 (-0.01, 0.01) |  |
| A mix of PAT and BAT | 9 | -0.00 (-0.00, 0.00) |  |
| **The purpose of laparoscopy use** |  |  | 0.83 |
| DL | 5 | -0.00 (-0.01, 0.01) |  |
| TL | 4 | 0.00 (-0.01, 0.01) |  |
| A mix of DL and TL | 11 | -0.01 (-0.03, 0.01) |  |

PAT, Penetrating abdominal trauma; BAT, Blunt abdominal trauma; DL, Diagnostic laparoscopy; TL, Therapeutic laparoscopy; CI, confidence interval.

**Table S4 Subgroups analyses results of mortality**

| **Subgroup** | **Studies (n)** | **Risk Difference (95% CI)** | **P value between groups** |
| --- | --- | --- | --- |
| **Study design** |  |  | 0.77 |
| Prospective study | 3 | 0.00 [-0.05, 0.05] |  |
| Retrospective parallel control study | 13 | -0.01 (-0.02, 0.00) |  |
| Retrospective before-after study | 4 | -0.02(-0.06, 0.01) |  |
| **Injury mechanism** |  |  | 0.6 |
| PAT | 7 | -0.01 (-0.02, 0.01) |  |
| BAT | 5 | -0.02 (-0.05, 0.00) |  |
| A mix of PAT and BAT | 9 | -0.01 (-0.04, 0.01) |  |
| **The purpose of laparoscopy use** |  |  | 0.63 |
| DL | 5 | 0.00 (-0.03, 0.03) |  |
| TL | 7 | -0.02 (-0.06, 0.02) |  |
| A mix of DL and TL | 9 | -0.01 (-0.04, 0.01) |  |

PAT, Penetrating abdominal trauma; BAT, Blunt abdominal trauma; DL, Diagnostic laparoscopy; TL, Therapeutic laparoscopy; CI, confidence interval.

**Table S5 Subgroups analyses results of complications**

| **Outcome** | **Subgroup** | **Studies (n)** | **Risk Difference (95% CI)** | **P value between groups** |
| --- | --- | --- | --- | --- |
| Wound infection | **Study design** |  |  | 0.95 |
|  | Prospective study | 3 | -0.04 (-0.20, 0.11) |  |
|  | Retrospective parallel control study | 10 | -0.04 (-0.08, -0.01) |  |
|  | Retrospective before-after study | 4 | -0.03 (-0.08, 0.01) |  |
|  | **Injury mechanism** |  |  | 0.32 |
|  | PAT | 6 | -0.07 (-0.15, 0.02) |  |
|  | BAT | 4 | -0.04 (-0.10, 0.02) |  |
|  | A mix of PAT and BAT | 7 | -0.01 (-0.03, 0.00) |  |
|  | **The purpose of laparoscopy use** |  |  | 0.80 |
|  | DL | 5 | -0.03 (-0.10, 0.04) |  |
|  | TL | 3 | -0.05 (-0.11, 0.00) |  |
|  | A mix of DL and TL | 10 | -0.03 (-0.06, -0.00) |  |
| Intra-Abdominal Abscess | **Study design** |  |  | 0.75 |
|  | Prospective study | 3 | 0.01(-0.04, 0.06) |  |
|  | Retrospective parallel control study | 9 | -0.01 (-0.02, 0.01) |  |
|  | Retrospective before-after study | 3 | -0.02 (-0.07, 0.03) |  |
|  | **Injury mechanism** |  |  | 0.39 |
|  | PAT | 5 | 0.00 (-0.02, 0.02) |  |
|  | BAT | 3 | -0.03 (-0.07, 0.01) |  |
|  | A mix of PAT and BAT | 7 | -0.01 (-0.03, 0.02) |  |
|  | **The purpose of laparoscopy use** |  |  | 0.63 |
|  | DL | 5 | 0.00 (-0.03, 0.03) |  |
|  | TL | 3 | -0.02 (-0.05, 0.01) |  |
|  | A mix of DL and TL | 8 | -0.01 (-0.03, 0.02) |  |
| Pneumonia | **Study design** |  |  | 0.14 |
|  | Prospective study | 3 | 0.00 (-0.06, 0.06) |  |
|  | Retrospective parallel control study | 6 | -0.02 (-0.04, 0.01) |  |
|  | Retrospective before-after study | 3 | -0.04 (-0.05, -0.03) |  |
|  | **Injury mechanism** |  |  | 0.23 |
|  | PAT | 5 | -0.01 (-0.04, 0.02) |  |
|  | BAT | 1 | 0.00 (-0.15, 0.15) |  |
|  | A mix of PAT and BAT | 6 | -0.04 (-0.05, -0.03) |  |
|  | **The purpose of laparoscopy use** |  |  | 0.48 |
|  | DL | 5 | -0.03 (-0.07, 0.02) |  |
|  | TL | 1 | 0.00 (-0.06, 0.06) |  |
|  | A mix of DL and TL | 7 | -0.04 (-0.05, -0.02) |  |
| Thromboembolism | **Study design** |  |  | 0.76 |
|  | Prospective study | 3 | 0.00 (-0.05, 0.05) |  |
|  | Retrospective parallel control study | 6 | -0.00 (-0.02, 0.01) |  |
|  | Retrospective before-after study | 3 | 0.00 (-0.00, 0.00) |  |
|  | **Injury mechanism** |  |  | 1.00 |
|  | PAT | 5 | 0.00 (-0.02, 0.02) |  |
|  | BAT | 1 | 0.00 (-0.15, 0.15) |  |
|  | A mix of PAT and BAT | 6 | -0.00 (-0.00, 0.00) |  |
|  | **The purpose of laparoscopy use** |  |  | 0.63 |
|  | DL | 5 | 0.00 (-0.03, 0.03) |  |
|  | TL | 2 | -0.01 (-0.02, 0.01) |  |
|  | A mix of DL and TL | 6 | 0.00 (-0.00, 0.00) |  |
| Bowel Obstruction or Ileus | **Study design** |  |  | 0.37 |
|  | Prospective study | 3 | 0.00 (-0.05, 0.05) |  |
|  | Retrospective parallel control study | 7 | -0.07 (-0.16, 0.02) |  |
|  | Retrospective before-after study | 4 | -0.02 (-0.08, 0.03) |  |
|  | **Injury mechanism** |  |  | 0.62 |
|  | PAT | 6 | -0.07 (-0.18, 0.03) |  |
|  | BAT | 3 | -0.01 (-0.14, 0.13) |  |
|  | A mix of PAT and BAT | 5 | -0.02 (-0.06, 0.02) |  |
|  | **The purpose of laparoscopy use** |  |  | 0.75 |
|  | DL | 5 | -0.03 (-0.08, 0.02) |  |
|  | TL | 2 | -0.06 (-0.15, 0.02) |  |
|  | A mix of DL and TL | 8 | -0.03 (-0.08, 0.02) |  |

PAT, Penetrating abdominal trauma; BAT, Blunt abdominal trauma; DL, Diagnostic laparoscopy; TL, Therapeutic laparoscopy; CI, confidence interval.

**Table S6 The sensitive analysis results using fixed effects model**

| **Outcome or Subgroup** | **Studies** | **Participants** | **Statistical Method** | **Effect Estimate** | **P value** | **I2** |
| --- | --- | --- | --- | --- | --- | --- |
| Missed injury | 19 | 5329 | Risk Difference (M-H, Fixed, 95% CI) | -0.00 [-0.01, 0.00] | 0.46 | 4% |
| Mortality | 20 | 4689 | Risk Difference (M-H, Fixed, 95% CI) | -0.03 [-0.05, -0.02] | <0.0001^*^ | 38% |
| Wound infection | 17 | 4471 | Risk Difference (M-H, Fixed, 95% CI) | -0.03 [-0.04, -0.02] | <0.0001^*^ | 46% |
| Abscess | 15 | 1339 | Risk Difference (M-H, Fixed, 95% CI) | -0.01 [-0.03, 0.01] | 0.21 | 0% |
| Pneumonia | 12 | 3435 | Risk Difference (M-H, Fixed, 95% CI) | -0.04 [-0.05, -0.02] | <0.00001^*^ | 0% |
| Thromboembolism | 12 | 3931 | Risk Difference (M-H, Fixed, 95% CI) | -0.00 [-0.01, 0.00] | 0.61 | 0% |
| Bowel obstruction or ileus | 14 | 3592 | Risk Difference (M-H, Fixed, 95% CI) | -0.06 [-0.07, -0.05] | <0.00001^*^ | 70% |
| Length of stay | 13 | 3543 | Mean Difference (IV, Fixed, 95% CI) | -3.25 [-3.37, -3.13] | <0.00001^*^ | 98% |
| Procedure time | 10 | 749 | Mean Difference (IV, Fixed, 95% CI) | -18.41 [-22.13, -14.70] | <0.00001^*^ | 84% |
| Re-operation | 6 | 1173 | Risk Difference (M-H, Fixed, 95% CI) | -0.03 [-0.04, -0.01] | 0.001 | 71% |

^*^Represents statistical significance;

MH, Mantel-Haentszel; IV, Inverse variance; CI, Confidence interval.

**Table S7 The pooled analysis results of high-quality studies**

| **Outcome or Subgroup** | **Studies** | **Participants** | **Statistical Method** | **Effect Estimate** | **P value** | **I^2^** |
| --- | --- | --- | --- | --- | --- | --- |
| Missed injury | 10 | 1253 | Risk Difference (M-H, Random, 95% CI) | 0.00 [-0.01, 0.01] | 0.97 | 0% |
| Mortality | 13 | 1386 | Risk Difference (M-H, Random, 95% CI) | -0.01 [-0.02, 0.00] | 0.28 | 0% |
| Wound infection | 10 | 1180 | Risk Difference (M-H, Random, 95% CI) | -0.05 [-0.08, -0.01] | 0.01^*^ | 42% |
| Abscess | 10 | 757 | Risk Difference (M-H, Random, 95% CI) | -0.00 [-0.02, 0.01] | 0.60 | 0% |
| Pneumonia | 7 | 449 | Risk Difference (M-H, Random, 95% CI) | -0.02 [-0.04, 0.01] | 0.21 | 0% |
| Thromboembolism | 7 | 945 | Risk Difference (M-H, Random, 95% CI) | -0.00 [-0.02, 0.01] | 0.46 | 0% |
| Bowel obstruction or ileus | 8 | 568 | Risk Difference (M-H, Random, 95% CI) | -0.02 [-0.06, 0.01] | 0.22 | 31% |
| Length of stay | 8 | 463 | Mean Difference (IV, Random, 95% CI) | -3.29 [-4.55, -2.02] | <0.00001^*^ | 62% |
| Procedure time | 7 | 420 | Mean Difference (IV, Random, 95% CI) | -15.27 [-25.15, -5.39] | 0.002 ^*^ | 48% |
| Re-operation | 6 | 1173 | Risk Difference (M-H, Random, 95% CI) | -0.02 [-0.04, 0.01] | 0.23 | 71% |

^*^Represents statistical significance.

MH, Mantel-Haentszel; IV, Inverse variance; CI, Confidence interval.
